# TrustWalker: An Efficient Trust Assessment in Vehicular Internet of Things (VIoT) with Security Consideration

**DOI:** 10.3390/s20143945

**Published:** 2020-07-16

**Authors:** Muhammad Sohail, Rashid Ali, Muhammad Kashif, Sher Ali, Sumet Mehta, Yousaf Bin Zikria, Heejung Yu

**Affiliations:** 1 School of Computer Science and Communication Engineering, Jiangsu University, Zhenjiang 212013, China; engrsohailaslam@gmail.com (M.S.); 5103160327@stmail.ujs.edu.cn (S.A.); 5103160334@stmail.ujs.edu.cn (S.M.); 2 Faculty of Engineering Science, Technology, and Management, Ziauddin University, Karachi 74600, Pakistan; 3 School of Intelligent Mechatronics Engineering, Sejong University, Seoul 05006, Korea; rashidali@sejong.ac.kr; 4 School of Engineering, Macquarie University, Sydney NSW 2109, Australia; Muhammad.kashif@hdr.mq.edu.au; 5 Department of Information and Communication Engineering, Yeungnam University, Gyeongsan-si 38541, Korea; 6 Department of Electronics and Information Engineering, Korea University, Sejong 30019, Korea

**Keywords:** IoT, VIoT, TrustWalker, trust enhanced routing, trust modelling

## Abstract

The Internet of Things (IoT) is a world of connected networks and modern technology devices, among them vehicular networks considered more challenging due to high speed and network dynamics. Future trends in IoT allow these inter networks to share information. Also, the previous security solutions to vehicular IoT (VIoT) much emphasize on privacy protection and security related issues using public keys infrastructure. However, the primary concern about efficient trust assessment, authorized users malfunctioning, and secure information dissemination in vehicular wireless networks have not been explored. To cope with these challenges, we propose a trust enhanced on-demand routing (TER) scheme, which adopts TrustWalker (TW) algorithm for efficient trust assessment and route search technique in VIoT. TER comprised of novel three-valued subjective logic (3VSL), TW algorithm, and ad hoc on-demand distance vector (AODV) routing protocol. The simulated results validate the accuracy of the proposed scheme in term of throughput, packet drop ratio (PDR), and end to end (E2E) delay. In the simulation, the execution time of the TW algorithm is analyzed and compared with another route search algorithm, i.e., Assess-Trust (AT), by considering real-world online datasets such as Pretty Good Privacy and Advogato. The accuracy and efficiency of the TW algorithm, even with a large number of vehicle users, are also demonstrated through simulations.

## 1. Introduction

**Figure 1:**
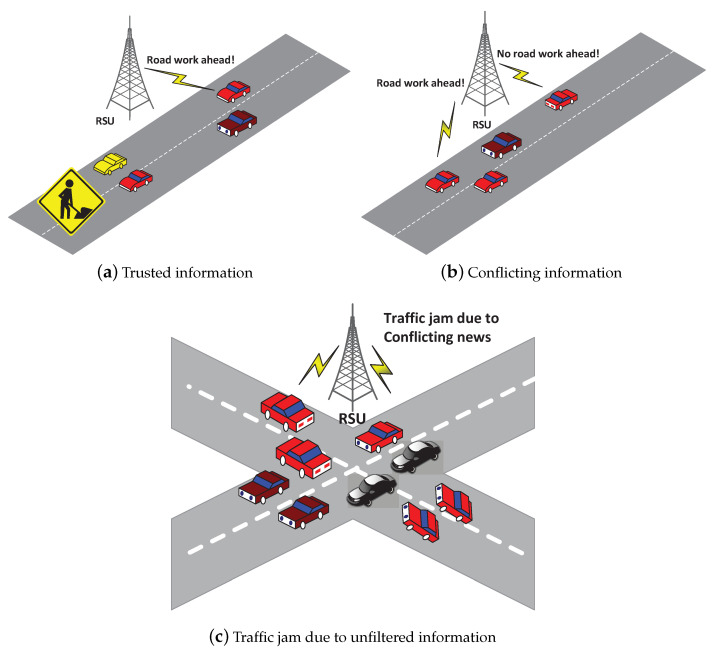
Traffic monitoring results in VIoT (**a**) Result of trust information which makes vehicles divert timely. (**b**) Conflicting news from two vehicles. (**c**) Result of unfiltered information from untrusted vehicles.

Vehicular Internet of Things (VIoT) network is a special wireless network where vehicles are equipped with digital computers and multiple sensors. New technologies and communication standards such as IEEE 802.11p or dedicated short-range communication (DSRC) allow vehicles to share information. VIoT differs from mobile networks in the sense that they do not have limited energy and storage capacity. Also, IoT technologies connect physical devices and share information using different wireless technologies [1,2]. According to one of the Cisco reports, there will be 400 million GB of data generated by 300 million passenger vehicles with wireless enabled devices in the near future [3]. For safety purposes, the data generated by these vehicles in VIoT must be genuine. Vehicular networks, as one of the promising applications in IoT, have changed the direction of travel plans, road safety, and entertainment. However, these networks can face serious security problems due to high speed, adversary attacks, and open nature of the network [4]. The adversaries can impede vehicular services and interrupt the whole traveling. For example, since the vehicles are driven by humans, there might be a chance of selfishness, and therefore vehicle performance is not equal. Meanwhile, the disseminated information from these vehicles in the VIoT need to be trustworthy, and this important issue should be borne in mind. If the trustworthiness of the particular vehicle is unknown, we can face the scenarios as depicted in Figure 1. In Figure 1a, a vehicle makes subjective assessment using on-board sensors that there is worksite ahead, so it informs the road-side unit (RSU). If the RSU knows that this information is received from the trusted vehicle, it will broadcast this information. In Figure 1b, the RSU gets the conflicting news about road divergence. If the RSU is unable to filter the received information as genuine or not, we can have a scenario as depicted in Figure 1c. In addition, previously proposed schemes based on public key infrastructure (PKI) in VIoT require central cloud services. Neither, it can be solved by any privacy-preserving scheme to authenticate the legitimacy of shared data [5,6]. Instead, incorporating trust can help us in building reputation system between unknown vehicle users and to evict malicious ones within its transmission range [7,8].

**Figure 2:**
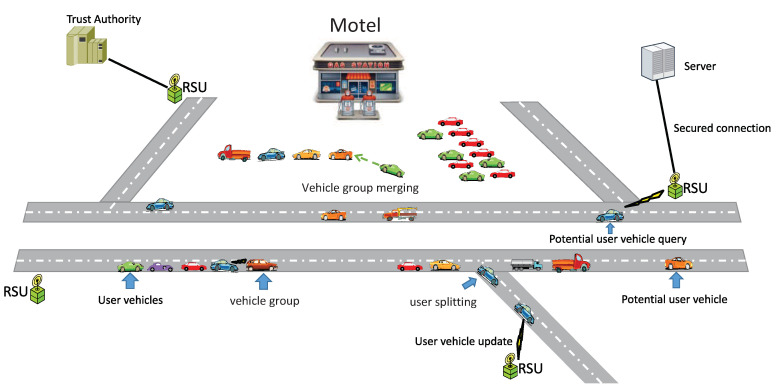
A possible scenario in future vehicular Internet of Things.

These new paradigms can be a unique opportunity for news sharing, emergency reports, and road safety for the connected vehicles in the VIoT technologies [1,2]. Some of the interesting use cases in vehicular internet of things can be found at (https://www.biz4intellia.com/blog/iot-applications-in-automotive-industry/)-(https://blog.westerndigital.com/top-9-iot-use-cases/). A possible scenario for information sharing among communicated devices in the future vehicular network is depicted in Figure 2. Meanwhile, the disseminated information from these integrated devices such as vehicles or RSUs needs to be trustworthy. If the false alarm and security attacks [4,7] remains undetected, then there is a chance of traffic jam or even worse false information dissemination.

In addition, many future promising applications in ITS can be supported by a hybrid structure as described in [8,9]. This hybrid structure contains trusted authorities (TA), RSUs, and vehicles on the road. However, it can be a crucial task to provide this central architecture everywhere on the whole journey. Generally, vehicles on the road may travel in close proximity by sharing different services among them. When a requesting vehicle wants to relay a message to the target vehicle, the question here is how to ensure the trustworthiness of the connecting vehicles as a truster and trustee in this VIoT. One solution is to incorporate trust management ideas among neighbor vehicles [10], which allows them to authenticate each other based on their trust score and finally to evict the malicious ones. Before relaying or receiving some traffic and safety information, a vehicle must know the credibility of the targeted vehicle. In this situation, a user vehicle establishes an efficient trust assessment and ignore misleading information sent by adversaries.

This study proposes a trust enhanced on-demand routing (TER) scheme, which takes inherent advantages of the TW algorithm for efficient trust assessment and an ad hoc on-demand distance vector (AODV). The proposed TER scheme uses the three-valued subjective logic (3VSL) and Bayesian network theory. 3VSL, which is a kind of advanced probabilistic logic, gives an accurate trust assessment result in the final computed opinion [11,12]. This feature urges us to use 3VSL in the proposed scheme because it defines trust as a threefold event by considering positive, negative, and neutral events. The neutral state expresses the posterior uncertainty which exists in trust generated by vehicles during an interaction. Prior uncertainty remains the same in most of the probabilistic trust models. Furthermore, the TW algorithm represents the vehicular network topology in the form of a matrix using 3VSL operations. The performance is analyzed to validate the accuracy of the proposed TER in terms of throughput, packet loss, and end-to-end (E2E) delay. Additionally, the execution time of the TW algorithm is analyzed by comparing it with another route search algorithm called Assess-Trust (AT) [13]. The execution times of the TW and AT schemes are evaluated by using two real-world on-line datasets i.e., Advogato and Pretty Good Privacy (PGP).

### Research Contributions

- First, based on our trust model, we propose on-demand trust enhanced routing (TER) among vehicles in VIoT, which extends trust-based source routing mechanisms by combining with the TW algorithm. Our proposed TER scheme predicts the next route-finding and efficient route discovery based on the current node threshold trust value and the TW algorithm for transmitting the information.
- Second, for the performance evaluation of the proposed TER, we compare it with the performance of two other routing protocols even though they do not have trust mechanism among communicating nodes. One is an authenticated anonymous secure routing (AASR) scheme that uses a cryptographic operation, and the other is standard AODV routing. Based on the analytical results, it is shown that the proposed TER is a promising approach in enhancing packet delivery, minimizing E2E delay, and resisting against malicious threats.
- Third, we argue that the proposed TER can resist certain malicious attacks by the continuous update of route trust by taking the latest interactions among peers. The TER helps us to cope with malicious attacks, e.g., black hole and zigzag.
- Finally, taking advantage of the TW algorithm, we can efficiently assess trust in large scale vehicular networks. Therefore, it can be seen that the proposed TW algorithm can also be applicable to interpersonal trust assessment in online social networks.

The rest of the paper is organized as follows: Section 2 explains the related work in two parts, i.e., a brief history of the proposed trust models and trust-based routing schemes in VIoT. In Section 3, we define a system model and trust assumptions along with the design goals for the proposed scheme. In Section 4, fundamentals on the trust model for the proposed scheme are described in details. Section 5 expresses the framework for the proposed TER. Section 6 provides details about the TW algorithm and its related operations in the TER. In Section 7, the route maintenance operations related to route error, route hand-off, and node mobility are explained. In Section 8, the simulation setup is carried out, and results are obtained. The conclusions are provided in Section 9.

## 2. Related Work

The application of the fifth-generation (5G) networks to VIoT promotes the interaction of V2X technology. In this scenario, the authenticity and credibility of disseminated information by integrated participants in the V2X technology is very important [2,14]. Furthermore, most of the existing trust management schemes in vehicular networks are based on public key infrastructure, batch authentication, and cloud-based trust management [15,16]. The author in [17] relied on a cryptographic routing operation. Rasool et al. proposed multi-channel operational medium access control protocols for vehicular environment [18]. Although some of these schemes are good enough to solve privacy preserving and authentication problems but fail to highlight the uncertainty issue and efficient trust assessment among unknown participants, e.g., unknown vehicles on the road. Therefore, a trust assessment mechanism among communicating vehicles is really required. A trust management system allows participants on whom they should trust or not. We summarized some of the research work related to our proposed scheme into two parts, first is the trust model in vehicular networks, and second is secure routing in vehicular networks.

### 2.1. Trust Models in Vehicular Networks

Only a few trust models were defined in vehicular networks for honest information sharing. Here, we will summarize them and highlight their drawbacks. Although a lot of research regarding the security and privacy of vehicles has been conducted, it is mostly based on the security infrastructure or certificate distribution mechanisms by a central agent. In this study, however, we focus on a trust model that does not entirely rely on a central agent and the peers may establish trust relationship based on direct-indirect interactions and the short history of recent behaviors by forming VIoT.

#### 2.1.1. Entry-Oriented Trust Models

Entity-oriented trust models can be divided into two categories: one proposed by Gerlach is based on sociological models [19], and the other suggested by Minhas et al. is a multi-faceted trust model [20]. In the first approach, trust intention is derived by using four input parameters as trust metrics. First, a situational decision that is based on the situation of an event irrespective of the trustee. Second, dis-positional trust irrespective of both trustee and the event situation, e.g., a kind of inherent optimism of the truster. Third, the central system-dependent trust mechanism in which both truster and trustees are included and provide assurance. Finally, a belief process is the assessment of the available data with a person’s own confidence in the final computed opinion, form the truster belief about a trustee in a specific context. However, one drawback of Gerlach’s work is the deficiency in the formalization of the architecture, i.e., a way to gather trust from different peers into a single opinion. The trust models such as multi-faceted features the quality of role-based and experience-based models as an evaluation standard to know vehicles trustworthiness. This method permits the vehicle to tell about others by sending the request, but limits the number of received reports. Perez and Marmol in [21] proposed TRIP and relied on reputation-based infrastructure to evict malicious nodes. In this method, the authors used three kinds of information sources, i.e., direct self-experience, a recommendation from neighbors, and recent history from a central authority. However, this idea fails to describe its genuineness in case of data sparsity and high speed of vehicles.

#### 2.1.2. Data-Oriented Trust Models

Data-oriented trust models come up as one of the great efforts as they evaluate the authenticity of the shared data among vehicles. Raya et al. in [22] proposed the data-oriented trust models for VIoT. In these trust models, a user is more interested in the trustworthiness of the shared data than that of a an important evaluation metric. Data-oriented trust models need cooperation among neighbor vehicles to assess the authenticity of the disseminated data. Huang et al. [23] proposed a voting-based system with different voting weights depending on its closeness to the place of the event. The authors in [24] proposed a deterministic approach to measuring the trust level of the received message by using the received signal strength (RSS) to calculate the distance and vehicle’s geolocation, i.e., position coordinate. Sohail et al. [13] proposed a multi-hop trust assessment scheme using a subjective logic framework among vehicle groups. This scheme describes the multi-hop trust assessment by considering prior and generated uncertainties in the shared data among vehicles. However, a major concern in data-oriented trust models is in latency and sparsity. As various sources will generate a large amount of data, a central entity is needed to perform operations efficiently. Subsequently, it increases the communication delay.

#### 2.1.3. Combined Trust Models

Combined trust models [25] overcome the drawbacks of the above mentioned trust models in VIoT. This trust model has an advantage to find the trustworthiness of both the shared data and the node itself. An attack-resistant trust (ART) scheme proposed in [26] calculates the trustworthiness of both data and shared messages among vehicles to resist against malicious attacks. The authors in [26] took functional and recommended trust to reach the final trust value, but they did not describe a data-sparsity problem in VANET. On the other hand, the author in [11] proposed centralized and distributive propositions so that it can handle a data-sparsity issue in high density vehicular network using a TW algorithm. The disseminated data is assessed first by taking direct interaction and recommendations, while the centralized authority (CA) acts to testify the authenticity of vehicles every time, which join or leave a network. Also, the storage server link with the central cloud is used to maintain vehicle social groups and manage trust regarding different vehicle behavior. In this way, the CA can have double check to evict the malicious vehicle in the network.

### 2.2. Secure Routing in Vehicular Networks

Routing protocols [27] act as a binding force among users in ad-hoc networks. Generally, we have two routing conditions, i.e., reactive and proactive routing protocols. Typically, the proactive routing protocol requires much bandwidth because paths among interacting users are maintained even if they are out of services. AODV is one of most popular approaches as the reactive routing protocol for mobile ad hoc networks because of its on-demand feature [28], which means that a node will only perform routing operations when it wants to discover a routing path towards other nodes for communication.

Moreover, the authors in [29] used real time traffic monitoring information with the help of GPS map and Sumo simulators as an enhanced version of AODV. Bravo et al. in [30] used the optimized reactive AODV routing over virtual nodes to gain some promising route results. Furthermore, some researchers used a complex cryptographic processing operation called authenticated anonymous secure routing in wireless ad hoc networks combined with AODV [17]. The authors in [31,32] proposed reactive and multi-path routing for MANET based on fuzzy logic and Markov chain phenomena. Li et al. in [33] proposed trust-based secure routing using subjective logic. Although the authors gain some promising results in above mentioned schemes, we argue that subject logic [34] is a more powerful probabilistic tool to highlight uncertainty and suspicious factor of unknown participants. Based on the existing work, we argue that the proposed TER secure routing is also suitable in this environment. The TER using the TW algorithm efficiently searches routes and solves massive trust assessment problem with uncertainty consideration.

## 3. System Model, Trust Model and Design Goals

### 3.1. System Model

A typical ITS scenario with a central authority, server, RSUs, and vehicles equipped with digital computers and on-board sensors is under consideration as shown in Figure 2. In particular, we consider a vehicular network topology, in which an individual user vehicle wants to assess trust of all other users vehicle within its transmission range. It is assumed that a centralized architecture is adopted to initialize the network and maintain a brief history of the vehicles, i.e., an opinion table and trust score. After the initial setup, the vehicles can authenticate each other by using the proposed scheme to reduce extra routing overhead, bandwidth, and time. The role of each network entity is as follows:

**TA**: A TA has a very significant role regarding registration of all RSUs, servers, and vehicles. Also, it has sufficient storage space for data servers, so it can maintain all evaluations records and a brief history of vehicles.

**RSUs**: RUSs communicate with vehicles through wireless channels such as base stations in cellular networks. The RSUs can be the first step to the network initialization and baseline for the first step towards security.

**Vehicles**: Vehicles on the road can be considered to be mobile nodes equipped with the on board unit (OBU). The vehicles within the specified transmission range can use the proposed TER scheme to search the routes among neighbor vehicles in VIoT. Among these vehicles, there are requesting and target vehicles.

### 3.2. Trust Model

We have made the following assumptions regarding the trustful roles of the entities in VIoT.

**TA**: A TA, which a central agent of the whole system, is under strong physical protection and not affected by adversaries.

**RSU**: RSUs are connected to the server through a secure communication channel. They will tackle to adversaries and never disclose internal information. However, it might be possible that some strong adversaries can hack this or even deploy bogus RSU. Nevertheless, The TA can inspect and recover RSU at a high level, if detected to be compromised.

**Requesting Vehicle**: It is assumed that the requesting vehicle is honest when computing the trust of a target vehicle for relaying information. Meanwhile, it also concerns about the privacy of the target vehicle.

**Target Vehicle**: Because vehicles are driven by humans, there is a chance to be selfish for saving its resources and to drop messages deliberately. To minimize this effect, an opinion table is built on each vehicle which is maintained and updated by the TA.

### 3.3. Design Goals

Vehicular networks are challenging due to network dynamics and high speed. Having these challenges, it is not easy to accurately assess trust among vehicles. To cope with these challenges, the following design goals are desirable.

- *Accurate trust assessment*: Many malicious attacks can interrupt vehicular networks. During the aforementioned situation, a trust management scheme is urgently needed to rank good and bad vehicles.
- *The relay ratio improvement:* The target vehicle in VIoT may drop the information due to selfishness or other packet dropping attacks. In this situation, if a requesting vehicle randomly selects a target vehicle, then the relay ratio will be degraded. Therefore, we need an efficient trust assessment scheme to improve the relay ratio by selecting a reliable vehicle.
- *Robustness against adversaries*: The central security architecture should be capable of tackling adversaries, i.e., the collusion of vehicles to provide bogus feedback to RSU [35]. The central authority also makes sure that RSUs are not compromised in VIoT.

### 3.4. Adversary Model

Due to openness and high-speed, vehicular networks are more susceptible to security attacks. In VIoT most of the outside adversary may be tackled by central architecture. The inside malicious attacks are difficult to recognize and need a suitable trust algorithm to evict them. Following malicious attacks are considered in this paper.

- Simple Attack (SA): In this attack, an attacker manipulates other nodes to interrupt the normal communication going on by altering the route information and data forwarding packets.
- Black Hole Attack (BH): In the Black hole attack, an attacker first convinces the normal node by claiming that it has the shortest path to its desired destination. Once this normal node sends all packet to the attacker and after getting all packets, it drops all packets, and the normal node becomes a victim. The attacker is difficult to recognize and remains active in the system.
- Zigzag (On-and-off) Attack (ZA): In this attack, the malicious nodes may act good and bad with changing alternative behavior. The malicious node on for a few seconds to send some packets and then suddenly gets off and drops the packets due to many selfish reasons.

## 4. Trust Model

We describe our trust model in two parts. The first is to calculate node trust with the help of direct and indirect trust using the Bayesian and subjective logic framework. The second is to calculate the path trust, which is evaluated by combining the trust values of all the vehicle users along a path with the efficiency of the TW algorithm.

### 4.1. Classification of Trust Types

**(a) Vehicle historical trust:** A brief history of vehicle trust can be taken from the central system. However, when two vehicles interact, we used data packet forwarding (DFR) and control packet forwarding ratio (CFR) as trust factors. Also, we assign weights to these trust factors to monitor the overall historical trust of the targeted vehicle.

**(b) Vehicle current trust:** A vehicle current trust is evaluated by the latest interactions among vehicles to predict future vehicle behavior using Bayesian network theory. It is computed by the latest interactions among vehicles to predict future vehicle behavior using Bayesian network theory. It is computed as a direct trust assessment based on physical vehicle interactions. Meanwhile, to accurately assess trust among vehicles, recommended or indirect trust is assessed with the help of novel 3VSL, which also highlights the generated uncertainty of an event. The prior uncertainty of an event remains fix or unchanged.

**(c) Route trust:** We choose to find the credibility of route in term of packets forwarding, and denoted as route trust value (RouteTV). When TW starts to discover a route, it must assure selected route authenticity. The route trust is calculated by combining the trust of intermediate nodes [36]. Also, the total route must be higher than the threshold level.

### 4.2. Direct Trust

In evaluating direct trust we used sliding window mechanism to take the latest interactions first. The latest event E can be divided into *t* time periods, i.e., $E=[E_{1},E_{2},E_{3},\cdots ,E_{t}]$. It is supposed that in each event $E_{i}$, node *x* can monitor $n_{i}$ times the forwarding behavior of node *z*. The packet forwarding ratios of node *z* in time event $E_{i}$ is denoted by a vector $V_{n_{i}}=\left[V_{n_{1}},V_{n_{2}},V_{n_{3}},\cdots ,V_{n_{i}}\right]$. Here, we set threshold level (*L*), if the number of time for which packet forwarding ratio is higher then threshold (*L*). If the number of times of which packet forwarding ratio $m_{i}$ is below threshold then *L* is $n_{i}-m_{i}$. We adopted the Bayes theorem framework to reduce errors in computing the reputation of a targeted node. Reputation in Bayes theorem is calculated using the previous reputation level and likelihood function.

The Dirichlet distribution [37] has three parameters [α,β,γ], showing positive, negative, and neutral evidence, while the total sum of an event is not greater than 1. Dirichlet distributions are commonly used as prior distributions in Bayesian statistics. Here, the current reputation vector represents the posterior and the last reputation vector shows prior probability. The general form of Dirichlet distribution function for these two functions is $Dir\left(a,b\right)=x^{a-1}{\left(1-x\right)}^{b-1}/D\left(a,b\right)$, where D(a,b) in this equation is a normalization constant and ensure that the total probability is 1. We set $Dir\left(a,b\right)=\int_{a}^{b}x^{a-1}{\left(1-x\right)}^{b-1}dx$, where *x* implies [0, 1]. We assume that $R_{i}$ represents the value of the *i*th reputation evaluation vector according to the Dirichlet distribution, and then calculation of posterior probability is $R_{i}=\left(fi\left(x\right)\int_{a}^{b}fi\left(x\right)R_{i-1}dx\right)R_{i-1}$, where *a* and *b* are integration interval. In this paper, two mutually independent events that are satisfy by Dirichlet distribution are taken as whether the forwarding rate is higher than the threshold value L, then it can be obtained $fi\left(x\right)=C_{n_{i}}^{m_{i}}x^{m_{i}}{\left(1-x\right)}^{n_{i}-m_{i}}$. The reputation evaluation vector sequence of node x to z is $R_{n}=\left[R_{1},R_{2},R_{3},\cdots ,R_{t}\right]$. As mentioned above, if $R_{i-1}∼Dir\left(a_{i-1},b_{i-1}\right)$, then $R_{i}∼Dir\left(a_{i-1}+m_{i},b_{i-1}+n_{i}-m_{i}\right)$ then the values $a_{1}=m_{1},b_{1}=n_{1}-m_{1},\cdots ,a_{i}=a_{i-1}+m_{i},b_{i}=b_{i-1}+n_{i}-m_{i}$. Therefore, at beginning, i.e., $t_{0}$, we can set $a_{0}=b_{0}=1,R_{0}∼beta\left(1,1\right)$ where beta(α,β) denotes a beta distribution. One of the trust metrics $t_{d_{i}}$ in $E_{i}$ time period can be computed and updated by using the expectation of beta distribution. The formula given as follow:

(1) $$t_{d_{i}}=\frac{a_{i}}{a_{i}+b_{i}},$$

for i=1,2,3,⋯. The direct trust of node *z* concerning node *x* observation is also affected by the amount of packet forwarded and interaction time. Therefore we use total packets forwarded and time attenuation factors for the above scenario. Then we use the following equation to compute direct trust.

(2) $$dt_{xz}=\frac{\sum_{i=1}^{t}\rho^{t-i}M_{xz}^{i}}{M}t_{d_{i}},$$

where $\sum_{i=1}^{t}\rho^{t-i}\;\left(0<\rho <1\right)$ denotes the time attenuation function with a weighting (or forgetting) factor ρ, and $M_{xz}^{i}$ denotes the amount of packets forwarded by node *z* for node *x* during the time event $E_{i}$. As more packets are forwarded, the confidence of the obtained trust value increases.

### 4.3. Indirect/Recommended Trust

For computing indirect trust, we choose subjective logic probabilistic theory as an advantage to highlight uncertainty and misbehavior detection [38,39,40,41]. Random vehicular network topology and dynamic communication patterns involve reasonable uncertainty among concerning vehicles. To cope with it, we adopted a novel 3VSL framework [12]. 3VSL express evidence as ternary opinion space vector and uses Dirichlet distribution to model this opinion vector, which can be expressed as

(3) $$\begin{array}{cc} \; & w_{z}^{x}=\left[b_{z}^{x},\;d_{z}^{x},\;n_{z}^{x},\;e_{z}^{x}\right],\\ \; & b_{z}^{x}+d_{z}^{x}+n_{z}^{x}+e_{z}^{x}=1, \end{array}$$

where $w_{z}^{x}$ is a opinion vector and shows *x* direct opinion in *z*. The elements in opinion vector are $b_{z}^{x}$, $d_{z}^{x}$, $n_{z}^{x}$, and $e_{z}^{x}$. Here, $b_{z}^{x}$ and $d_{z}^{x}$ show solid evidence in favor and disfavor of an event to reach some conclusions, respectively. Neutral evidence includes prior $e_{z}^{x}$ and posterior uncertainty $n_{z}^{x}$, which neither supports positive or negative evidence. A vehicle may gain recommended trust if its subjective opinion is not enough to reach on solid conclusion.

#### 4.3.1. Consensus Combination

Let, $w_{y}^{x}$ and $w_{y}^{z}$, be x’s and z’s trust in y respectively. Then $w_{y}^{x}=\left[b_{y}^{x},\;d_{y}^{x},\;n_{y}^{x},\;e_{y}^{x}\right]$ and $w_{y}^{z}=\left[b_{y}^{z},\;d_{y}^{z},\;n_{y}^{z},\;e_{y}^{z}\right]$. Then consensus operation of x and z about trustworthiness of y is given by $w_{y}^{x,z}=w_{y}^{x}\Theta w_{y}^{z}=\left[b_{y}^{x,z},\;d_{y}^{x,z},\;n_{y}^{x,z},\;e_{y}^{x,z}\right]$. Four elements of $w_{y}^{x,z}$ are as follows:

(4) $$\begin{array}{cc} b_{y}^{x,z} & =\frac{b_{y}^{x}e_{y}^{z}+b_{y}^{z}e_{y}^{x}}{e_{y}^{x}+e_{y}^{z}-e_{y}^{x}e_{y}^{z}},\\ d_{y}^{x,z} & =\frac{d_{y}^{x}e_{y}^{z}+d_{y}^{z}e_{y}^{x}}{e_{y}^{x}+e_{y}^{z}-e_{y}^{x}e_{y}^{z}},\\ n_{y}^{x,z} & =\frac{n_{y}^{x}e_{y}^{z}+n_{y}^{z}e_{y}^{x}}{e_{y}^{x}+e_{y}^{z}-e_{y}^{x}e_{y}^{z}},\\ e_{y}^{x,z} & =\frac{e_{y}^{x}e_{y}^{z}}{e_{y}^{x}+e_{y}^{z}-e_{y}^{x}e_{y}^{z}}. \end{array}$$

The consensus operation Θ decreases uncertainty as it comes from both vehicles subjective experience about the particular vehicle.

#### 4.3.2. Discounting Combination

Assume two vehicles x and z where x trusts z, denoted by $w_{z}^{x}=\left[b_{z}^{x},\;d_{z}^{x},\;n_{z}^{x},\;e_{z}^{x}\right]$, for the purpose of judging the trustworthiness of y. In addition z has trust in y, denoted by $w_{y}^{z}=\left[b_{y}^{z},\;d_{y}^{z},\;n_{y}^{z},\;e_{y}^{z}\right]$. Vehicle x can then derive its trust in y by discounting z’s trust in y with x’s trust in z, denoted by $w_{y}^{x:z}=w_{z}^{x}△w_{y}^{z}=\left[b_{y}^{x,z},\;d_{y}^{x,z},\;n_{y}^{x,z},\;e_{y}^{x,z}\right]$. Four elements of $w_{y}^{x:z}$ are as follows:

(5) $$\begin{array}{cc} b_{y}^{x:z} & =b_{z}^{x}b_{y}^{z},\\ d_{y}^{x:z} & =b_{z}^{x}d_{y}^{z},\\ n_{y}^{x:z} & =1-b_{z}^{x}b_{y}^{z}-b_{z}^{x}d_{y}^{z}-e_{y}^{z},\\ e_{y}^{x:z} & =e_{y}^{z}. \end{array}$$

The discounting operation △ increases uncertainty as it comes from building one vehicle subjective opinion on other vehicle’s objective assessment.

## 5. Framework for Proposed TER

In our proposed framework, we have three stages. In the first stage, a basic routing protocol is operated among neighbor vehicles and nodes observation. At the second stage, nodes perform trust establishment operations such as trust recommendation, consensus, trust judgment, trusted routing operations, and trust update. In the third stage, vehicles can manage trust relationships using the proposed TER. The general procedure for a vehicle to join or leave the existing VIoT is as follows: In the beginning, most of the vehicles are uncertain towards each other, i.e., with less trust and high uncertainty. Therefore, at the start, vehicles are unable to fully trust or distrust a vehicle due to a lack of interactions. For example, a vehicle *A* wants to discover a path towards a vehicle *B* but yet unsuccessful as due to high uncertainty. At this stage, the vehicle *A* can use some basic routing operation to interact with the vehicle *B*, which helps to reduce its opinion towards the vehicle *B*. After the initialization of VIoT, each vehicle will have some positive or negative interactions towards each other, and uncertainty will be reduced.

At network initialization vehicles use the TER scheme, which is based on the trust model to perform routing operations. In our scheme to reduce extra routing overhead, vehicles can use a trust recommendation protocol by exchanging trust scores with their routing request. A receiving vehicle will use their trust scores and combine all the recommended and observed opinions into the final one. A vehicle will follow the route discovery and maintenance operations proposed in the TER scheme. At network start, how a vehicle will join or leave the VIoT, depends on the proposed algorithm and security requirements.

### 5.1. Routing Table Extension

In the proposed TER, the trust repository counter is added to the original AODV routing table, as given in Table 1. Once the initial setup among vehicles in VIoT is established, vehicles can use the TER scheme, which is based on the above mentioned framework.

### 5.2. Trust Judging and Update Rules

In our scheme, we choose the threshold level to be 0.5, and intermediate nodes actions with respect to this threshold are given in Table 2. We can use a high threshold value if the system is sensitive to security requirements.

- If the belief of neighbor vehicle *x* in *z* is greater than the given threshold, then it will trust vehicle *z* and vise versa.
- If the uncertainty of vehicle *x* in vehicle *z* is greater than 0.5, *x* ask for a digital signature verification from vehicle *z* and waits for it. If *x* successfully verifies *z*’s signature then *x* will start communicating with vehicle *z*.
- If two neighbor vehicles had positive interaction, then the trust repository is updated by increment the trust in the correspondent vehicle, and the inverse is true also.
- If two vehicles have any uncertainty between each other, then we use $w_{z}^{x}=\left(0,0,0,u\right)$. Here, *u* is the maximum uncertainty from vehicle *x* to vehicle *z*. It is because the two vehicles never interacted by the given expiry time of 5 s.

### 5.3. Information Exchange Protocol

**Figure 3:**
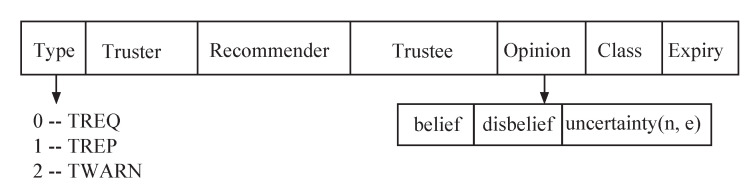
Information exchange format among neighbor vehicles using three kinds of route information.

Previously, proposed trust models rarely considered trust recommendation exchange protocol among these distributed vehicle users in VIoT. However, this information exchange helps us to reduce extra routing overhead between neighbor vehicles. This mechanism contains three types of messages, i.e., TREQ (Trust Request), TREP (Trust Reply), and TWARN (Warning), as can be seen in Figure 3. Generally, if a vehicle *x* wants to check the neighbor vehicle *y*’s trustworthiness, it will broadcast a trust request TREQ message. The format for TREQ is given in Figure 3, the truster type is filled with 0, and the trustee field filled with IP address of vehicle *y*. If one of *x*’s neighbor let say, *z* receives this broadcast message TREquation Vehicle *z* will reply by setting the type field to 1, and the opinion field is filled with the updated value from vehicle *y* to *z*. In this recommendation a vehicle can send route request or route reply to multiple vehicles in one routing packet.

The third type of message is TWARN that is used to report invalidation in standard reactive routing process by type field set to 2 after that neighbor vehicle can know about the trust warning.

## 6. Route Discovery by TW

In this section, we have used two algorithms, i.e., TW and authentication. TW, which is used to search for routes efficiently. The second algorithm is based on trust judgment rules given in Table 2. These two algorithms complete trusted route discovery.

### 6.1. Design of TW

The TW algorithm uses the Breadth-First Search (BFS) method to search routes more efficiently. We have taken vehicular topology with *n* users and form opinion matrix of n×n as follow

(6) $$OM=\left[\begin{array}{cccc} w_{11} & w_{12} & \cdots & w_{1n}\\ w_{21} & w_{22} & \cdots & w_{2n}\\ \vdots & \vdots & \ddots & \vdots \\ w_{n1} & w_{n2} & \cdots & w_{nn} \end{array}\right],$$

while opinion $w_{ij}$ shows *i*’s direct relation on *j*, similarly each element in OM shows direct relationship between each other. From user *i* (truster) point of view other elements can be shown in individual opinion vector as follow

(7) $$y_{i}^{k}={\left[\Omega_{i1}^{k},\;\Omega_{i2}^{k},\;\cdots ,\;\Omega_{ij}^{k},\;\cdots ,\;\Omega_{in}^{k}\right]}^{T},$$

where $\Omega_{ij}^{k}$ represents user *i*’s opinion on *j* as TW algorithm walks *k* hops on the network. The initial form of single element $\Omega_{i1}^{\left(1\right)}$ in opinion vector is represented as

(8) $$\Omega_{i1}^{\left(1\right)}=\left\{\begin{array}{c} w_{ij},\;if\;user\;i\;directly\;connects\;to\;j\\ u,\;uncertain\;opinion\;otherwise \end{array}\right.$$

where $w_{ij}$ shows direct relation from *i* to *j*. If *i* is not connected to *j* then it is replaced by uncertain opinion *u*. In this way, the TW algorithm update the individual vector in iterative manner by searching the network hop by hop fashion.

(9) $$y_{i}^{k}=OM^{T}y_{i}^{\left(k-1\right)}.$$

### 6.2. Operations in TW

**Figure 4:**
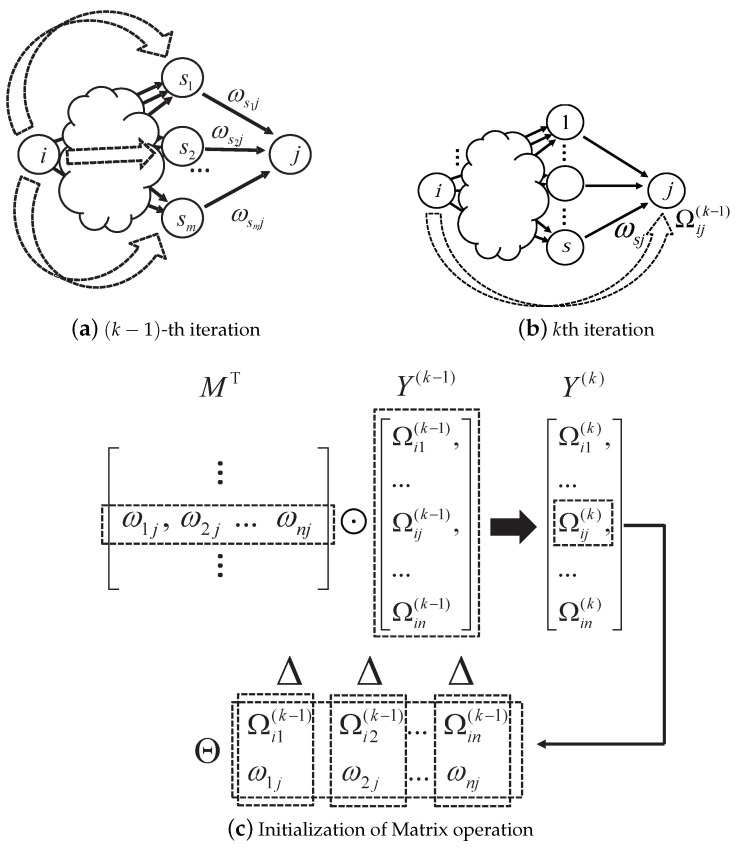
Matrix formation and initialization in TW route discovery.

The TW algorithm starts from user *i* and searches the network as depicted in Figure 4. Suppose the TW algorithm finds users that are not in his territory and (k−1)-hop away from user *i*. Among these users, we assume that user *j* is *k*-hop away from user *i*, and among these users, *m* are directly connected to *j*. We label these *m* users as $s_{1},s_{2},\cdots ,s_{m}$.

When the TW algorithm moves from the previous level to the next level, i.e., from the (k−1)th level to the *k*th level, it updates the corresponding opinion of the user *i*’s on user *j* as follows:

(10) $$\begin{array}{c} \Omega_{ij}^{k}=\Theta \left(△\left(\Omega_{is_{1}}^{\left(K-1\right)},w_{s_{1}j}\right),...,△\left(\Omega_{is_{m}}^{\left(K-1\right)},w_{s_{m}j}\right)\right)\\ \Omega_{ij}^{k-1}=\Theta \left(△\left(\Omega_{is_{1}}^{\left(K-2\right)},w_{s_{1}j}\right),...,△\left(\Omega_{is_{m}}^{\left(K-2\right)},w_{s_{m}j}\right)\right), \end{array}$$

The Equation (10) combines all *m* opinions that are computed as discounting $w_{s_{1}j}$ by $\Omega_{is_{l}}$, for all l=1,2,⋯,m. If $\Omega_{ij}^{k}\neq u$, i.e., the TW algorithm already had user *i*’s opinion on user *j* from the previous interaction. In this case, user *i*’s opinion on user *j* is replaced with $\Omega_{ij}^{k}$. In other words, only the opinions $\Omega_{is}^{k-1}$ where $w_{sj}\neq u$ are used in individual opinion vector updating. Finally, the TW operation multiplies matrix and individual vector $y_{i}^{k-1}$ to yield an updated vector $y_{i}^{k}$ as follows:

(11) $$\begin{array}{cc} y_{i}^{k} & =OM^{T}y_{i}^{\left(k-1\right)} \end{array}$$

(12) $$\begin{array}{cc} \; & =\left[\begin{array}{ccc} \Theta \left(△(\Omega_{i1}^{k-1},w_{11}),\right. & \cdots , & \left.△(\Omega_{in}^{k-1},w_{n1})\right)\\ \Theta \left(△(\Omega_{i1}^{k-1},w_{12}),\right. & \cdots , & \left.△(\Omega_{in}^{k-1},w_{n2})\right)\\ \; & \vdots \\ \Theta \left(△(\Omega_{i1}^{k-1},w_{1n}),\right. & \cdots , & \left.△(\Omega_{in}^{k-1},w_{nn})\right) \end{array}\right] \end{array}$$

(13) $$\begin{array}{cc} \; & ={\left[\Omega_{i1}^{k},\;\Omega_{i2}^{k},\cdots ,\;\Omega_{ij}^{k},\cdot ,\;\Omega_{in}^{k}\right]}^{T}. \end{array}$$

In a matrix-vector multiplication, we use the operations defined in 3VSL. Let us look at element $\Omega_{ij}^{k}$ in $y_{i}^{k}$, where i≠j. It is computed by multiplying vectors ${\left[\Omega_{i1}^{k-1},\Omega_{i2}^{k-1},...,\Omega_{in}^{k-1}\right]}^{T}$ and $\left[w_{1j},w_{2j},...,w_{nj}\right]$.

### 6.3. TW Algorithms

TW algorithm uses two nested loops, so each loop has a time complexity of $O\left(n\right)\times O\left(n\right)=O\left(n^{2}\right)$. If *H* is searching depth of network and H=K time complexity its will be $O(Kn^{2})$. It is much suitable for a highly dense network as compared to other route search algorithm, i.e., AT [11]. AT algorithm has high time complexity because it needs to search one to one user in network. However, this is not feasible for trust assessment in high-density VIoT. The time complexity in computing each user trustworthiness will be $O\left(\left(n-1\right)n^{k-1}\right)=O\left(n^{k}\right)$. Here, *k* shows the total searching depth in the network.

Algorithm 1 starts from line 3, defining a minimum number of hops. Line 4, iteratively search the number of hops on the network. In TW, we used two nestle loops in which line 5–14 updates the indirect opinion $\Omega_{ij}$, and lines 7–12 compute all opinions from $w_{sj}\neq u$. Line 8 shows *i*’s direct opinion on one of the sibling of *j*, i.e., *s*. At line 9 *i* discount *s*’s opinion on *j* to update $\Omega_{ij}^{\left(k\right)}$. Line 10 combine all previously computed opinion from $w_{sj}\neq u$.

$$\Theta (△\left(\Omega_{i_{1}}^{\left(K-1\right)},w_{1j}\right),..,△(\Omega_{i_{n-1}}^{\left(K-1\right)},w_{n-1j},△\left(\Omega_{i_{n}}^{\left(K-1\right)},w_{nj}\right).$$

Line 13 uses $\Omega_{ij}$ to updates corresponding elements in individual opinion vector. After updating All possible opinions at line 14, TW search next level.
**Algorithm 1:** Route Discovery by TWREQUIRE: A directed graph G with a truster *i* and the maximum searching level *H*ENSURE: $i^{\prime }s$ opinion on *J* where i≠j1. Initialize OM and $OV_{i}^{\left(1\right)}$ based on *G*2. k←13. **While** k<H **do**4. k←k+15. **for all** columns $C_{j}\in OM$ s.t. j≠i **do**6. $\Omega_{ij}^{k}\leftarrow U$7. for all direct opinion $w_{sj}\in C_{j}$ s.t. $w_{sj}\neq u$ **do**8. $\Omega_{is}^{\left(k-1\right)}\leftarrow y_{i}^{\left(k-1\right)}\left[s\right]$9. **if** $\Omega_{is}^{\left(k-1\right)}\neq u$ **then**10. $\Omega_{ij}^{k}\leftarrow \Theta \left(\Omega_{ij}^{K},\Delta \left(\Omega_{is}^{K-1},w_{sj}\right)\right)$11. **end if**12. **end for**13. $y_{i}^{\left(k\right)}\left[j\right]\leftarrow \Omega_{ij}^{\left(K\right)}$14. **end for**15. **end while**16. **return** $y_{i}^{k}$

The pseudo-code for user authentication is described in Algorithm 2. Here, we set the threshold with 0.5 for user authentication and make decisions to update $\Omega_{ij}^{k}$ according to the rules given in Table 2.
**Algorithm 2:** Authentication from user *i* to *j* in *k*-hop by TWExchange opinion about opinion matrix *i* with all neighbors of individual vector *j* using the TW information exchange protocol.;1. /* Verify the trustworthiness of i−j*/2. /* $\Omega_{ij}^{k}\neq u$ and judge the next step using conditions set in Table 1 */3. ***if*** $\Omega_{ij,b}^{k}\geq 0.5$4. trust i−j and forward RREQ/RREP5. ***elseif*** $\Omega_{ij,d}^{k}\geq 0.5$6. distrust i−j for expiry time7. ***elseif*** $\Omega_{ij,u}^{k}\geq 0.5$ request and verify digital certificate8. ***else***9. /* the confidence about trustworthiness is decreased*/request and verify *j*’s certificates, by default10. ***endif***

## 7. Route Maintenance

Route maintenance is the procedure by which a source node is able to detect the faults of the forwarding path towards the destination node. These faults can be due to some malicious attack pattern, with link broken event, and even below defined threshold trust level. In this scenario, the source node needs to reroute the information with an alternative route. In case of link broken event, i.e., the topology has changed, or the route trust does not meet the criteria so that it will trigger new trust judgment. Another thing Route maintenance assures the packet delivery within a specific time interval after a route cache crosses the maximum valid time, the packet will expire automatically.

### 7.1. Computation of Route Trust

Refer to the axiom [36]; the total is equal to the route trust values of intermediate nodes. The route trust at time *t* (denoted by RouteTV(t)) is computed by route search algorithm TW and it will be equal to combined trust values of the intermediate nodes.

### 7.2. Route Hand-Off

When the target node has detected that the current route trust is going to be un-trusted, a Flow-Hand-off message is generated by the target and propagated using FLOW-REQ message. After the source receives a Flow-Hand-off message, it chooses the best route to forward the received information.

**Figure 5:**
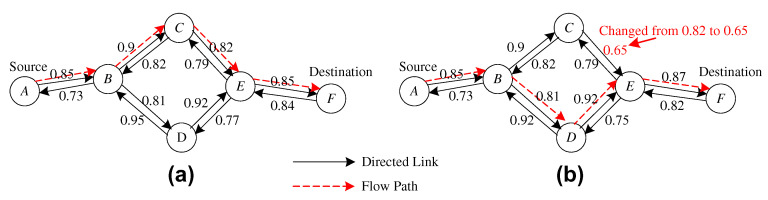
The Hand-off process: (**a**) the original route, from A to F; (**b**) the new route after the Hand-off process.

After detecting another best route that completes the minimum trust threshold requirement, the source node sends the FLOW-SETUP message. This FLOW-SETUP message is the same as it was sent the first time during route setup, except it has changed the path from source to target node. The Route Hand-off process is depicted in Figure 5. If the RouteTV (Route trust value) for the requirement of packet forwarding is above 0.5. In Figure 5a, the old route is along of nodes (i.e., A→B,→C,→E,→F,RouteTv=0.53) and if this route trust value RTv is about to un-trusted (RouteTVp=0.43), so the Route Hand-off is performed and the new route is now (i.e., A→B,→D,→E,→F,RouteTv=0.55).

### 7.3. Route Error

The target node generates route error request RERR by link layer feed back that is sent back to the source node to acknowledge link failure. Also, if a particular path remains unused for a specific period of time, then it will be considered invalid and removed from the source node cache.

### 7.4. Dealing with Node Mobility

Based on our proposed trust model, nodes could identify malicious or normal users along its path. The trust values among neighbor nodes can also be shared with the help of information exchange protocol in our scheme to reduce routing overhead [36]. At start generally, the uncertainty towards each other is normally high, still, when these nodes start to move and interact with each other, this uncertainty gradually decreases by positive or negative interaction, and trust mechanism is there for the entire network. For nodes with lower trust values the network does not send route query packets again, which reduces routing overhead and latency between end to end users. Also, we propose the latest trust interaction among neighbor nodes that comes along the last used route information along the path. Since vehicular network topology changes the time being to update the trust of the connection and time inform about Hand-off process.

A single node only stores recent interaction history and trust values of neighbor nodes, not all route-cache history as network topology in the vehicular network changes frequently. So a node is free to join or release the network any time. Also, if a particular path remains unused for a specific period of time, then it will be considered invalid and removed from the source node cache. Meanwhile, a node can learn and cache multiple routes to the target node using the extension of the TW algorithm. It allows that if the route changes rapidly, a node with multiple route cache to the target node can try to find alternatives in case if certain links get failed. This multiple route-cache also reduces overhead as it does not need to perform new route discovery every time the link failure is detected.

## 8. Simulation Setup and Results

In the experimental setup, we have considered a scenario in which neighbor vehicles make small social groups in VIoT. To evaluate trustworthy of the target vehicle for relay/receive information within this group, we have used NS-2.35 [42] network simulator. Performance analysis in terms of throughput, latencies, packet loss, is conducted to validate our proposed TER scheme. These simulations are performed using Intel Core-i3 and 4 GB SDRAM with 2.66 GHz Lenovo machine running on Ubuntu-12.04, 64-bit operating system. The basic parameters of our simulation are listed in Table 3.

### 8.1. Attack Pattern and Evaluation Metrics

To resist against different kinds of internal malicious attacks, the three routing protocols behave as follows, (1) AASR applies an initial complex cryptographic algorithm during initialization. (2) AODV takes no action against these attacks and forwards the packet as it gets. (3) TER can detect the malicious user via the updated trust score and get rid of the attackers. Furthermore, we have considered the zigzag and black-hole attacks as an adversary model.

Also, packet throughput, latency, and packet loss are evaluation metrics. First, the performance analysis is carried out among our proposed TER, Authenticated Anonymous Secure Routing AASR, and original Ad-hoc On-demand distance vector routing AODV using different vehicle mobility and increasing malicious nodes. In other performance analysis, TER and AODV are tested under black-hole and zigzag attack pattern. The time complexity analysis to validate the efficiency of TW is also carried out by considering two real-world data sets, i.e., (Advogato and PGP). Third, uncertainty analysis is also done that predicts the general behavior of vehicles during network initialization and setup.

### 8.2. Performance Analysis

#### 8.2.1. Test: 1 Packet Throughput, PDR, and E2E Delay with Different Vehicles Mobility and Increasing Malicious Nodes

**Figure 6:**
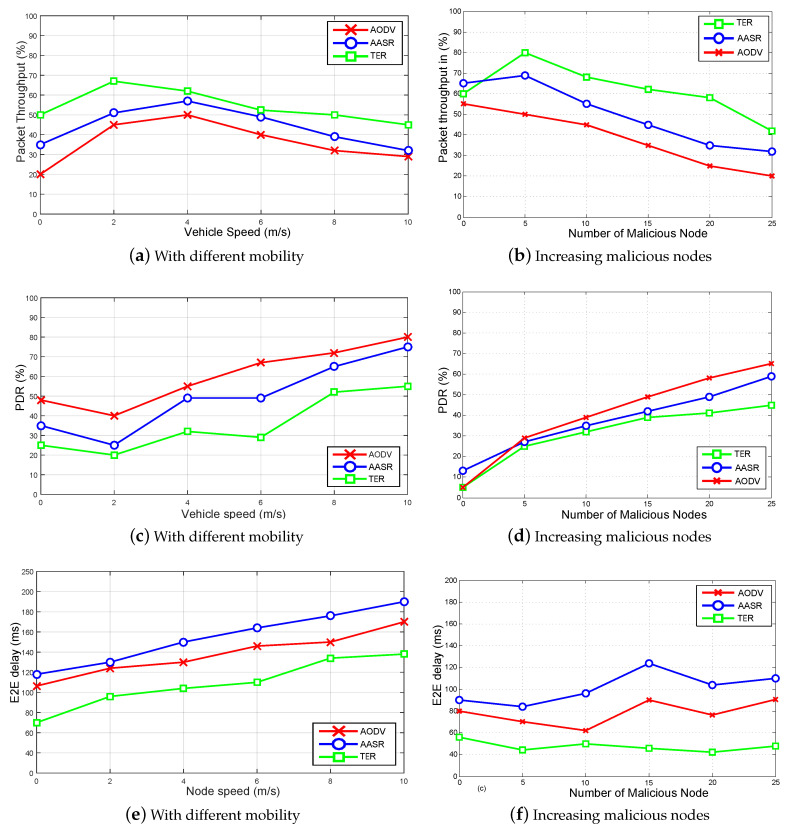
Packet throughput, PDR, and E2E delay with different vehicles mobility and increasing malicious nodes.

In this part, we first carried out performance analysis among our proposed scheme TER, original AODV, and AASR. Under different vehicles mobility and an increasing number of malicious nodes, the performance of these three schemes varies over time. From Figure 6, it is clear that TER outperforms the other two routing schemes, i.e., AASR and AODV. As vehicles reaches up to 10 m/s our scheme still have 50% of throughput, see Figure 6a. In second Figure 6b, as half of nodes become malicious (25 nodes) our scheme TER can achieves 45% packet throughput, AASR gives 35%, and AODV gives 30%. So it favors the TER scheme under these two scenarios.

Second, Packet loss is also an important metric to check any communication network performance. In Figure 6c, our proposed scheme has less packet loss drop. Since AODV takes no additional actions, so it has a high packet loss of 75% at V=10 m/s. AASR spend some time on initial cryptographic processing, so packet loss is 70% at V=10 m/s. TER minimizes this effect by using trusted information exchange among neighbor vehicles. TER has minimum packet loss of 55% at V=10 m/s. Also, when we have half of the nodes malicious, i.e., (25 nodes are malicious), the performance of these three schemes varies. AODV has 65% of packet loss, AASR has 55% of packet loss, and TER has 45% of packet loss, which is minimum among these schemes, see Figure 6d.

Third, E2E delay is a vital metric for measuring any network performance, especially for a delay-sensitive network such as VIoT. In this analysis, we compared our proposed scheme TER with AASR and AODV. AODV has E2E delay of 150 (ms) at V=10 m/s. However, AASR spends time in the route discovery and making some initial security processing, so it has a high E2E delay of 190 (ms) at V=10 m/s. TER minimizes this effect by using trusted information exchange among neighbor vehicles. TER has E2E delay of 130 (ms) at V=10 m/s, see Figure 6e. Similarly, in the presence of 50% malicious nodes, TER has a minimum E2E delay of 50 (ms). TER outperforms the other two routing schemes as AODV and AASR have E2E delay of 110 (ms) and 90 (ms) respectively, see Figure 6f.

#### 8.2.2. Test: 2 Packet Throughput, PDR, and E2E Delay under Black Hole and Zigzag Attacks

**Figure 7:**
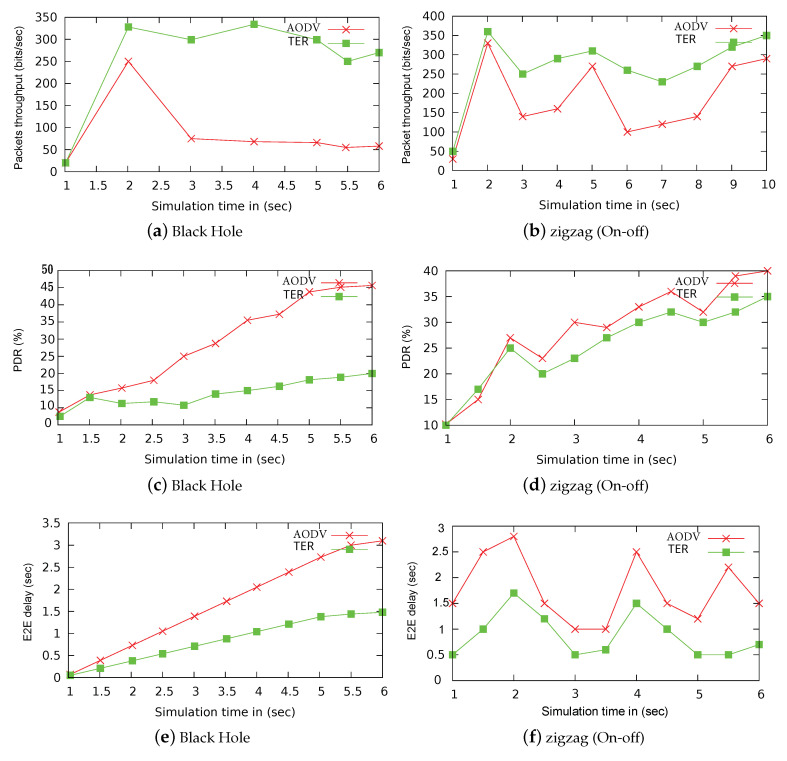
Packet throughput, PDR, and E2E delay under Black Hole and zigzag attacks.

In this part, first, we carried out a performance analysis between our proposed scheme TER and AODV. Under the malicious attacks, the performance of these schemes varies. From Figure 7, it is clear that TER outperforms original AODV schemes. At start of simulation our scheme achieves 350 (bits/s), while AODV gives 270 (bits/s), please refer Figure 7a. In Figure 7b, zigzag attacks frequently change its pattern as good or bad nodes, so our scheme enabling trust mechanism outperforms AODV and achieves better throughput. TER and AODV have an average throughput of 300 (bits/s) and 230 (bits/s) respectively under zigzag attack.

Second, under the black hole attack, as the start of the simulation, both schemes have lower packet loss ratio, after a few seconds, the packet loss of AODV is 45%, and our scheme has 20%, see Figure 7c. In Figure 7d, zigzag attacks frequently change its pattern as on-off, our scheme outperforms AODV scheme and achieves minimum packet loss. TER minimizes this effect using trust information exchange; after a few seconds, TER gives 30% of packet loss. However, the original AODV has no mechanism of trust-based information; it simply routes packets, so it gives a 50% packet loss ratio.

Third, at the start of the simulation, both schemes have less E2E delay, after a few seconds, the E2E delay of AODV is 3 (s), and TER has a delay of 1 (s), see Figure 7e. In Figure 7f, zigzag attack frequently changes its pattern as on-off, our scheme outperforms the AODV scheme and achieves minimum E2E delay. TER minimize this effect using trust information exchange; after a few seconds, TER gives 0.5 (s). The original AODV has no mechanism of trust-based information, and it simply routes the received packets, so increases E2E delay as 2 (s).

### 8.3. Execution Time

We analyzed the execution time in the route discovery phase with the help of two online software development communities, namely Pretty good privacy PGP and Advogato [43,44]. George’s opinion is used to build the honesty of Alice in the previously mentioned software development. This trustworthiness among connected users is categorized as given in Table 4. The execution time of these two software development communities is analyzed with AT [11] and TW algorithm.

In PGP and Advogato datasets, the edge weights are given in ordinal values. To convert these values into real numbers on a scale of 1, we used linear transformation techniques [45,46]. Table 5 lists enumeration of these datasets trust values to trust levels.

**Figure 8:**
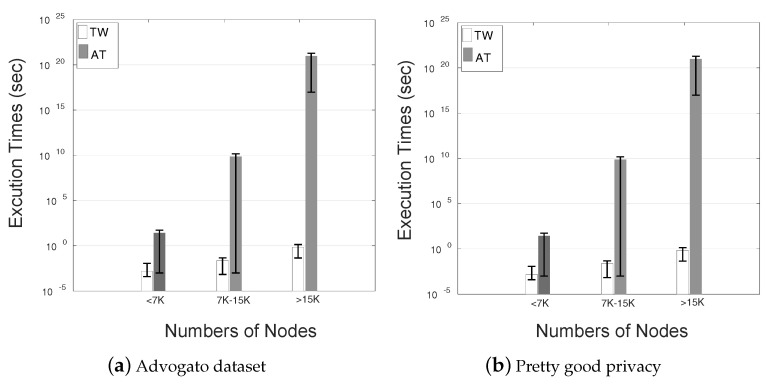
Execution time of AT and TW algorithm.

The accuracy of the AT and TW algorithm is checked in addressing the execution time of the trust assessment problem efficiently. We used Python 3.7 [47] on Window-7-64 bit operating system. We run these algorithms on the sub-graphs containing the edge from truster to trustees. The PGP and Advogato datasets are divided into three parts based on the users. The user’s category in Advogato and PGP are (<1K, 1K–3K, and >3K), (<7K, 7K–15K, and >15K). After that, we plot the execution time of AT and TW algorithms based on the above-mentioned user categories in Figure 8.

For highly dense network AT algorithm need to execute $O(n^{K})$ times to solve massive trust assessment problem, which corresponds to a larger searching depth and becomes extremely slow. On the other hand, TW is much efficient for trust assessment in VIoT, as execution time converges to $O(n^{2})$. Combining and discounting operation in TW is a little complex as compared to summation and multiplication. However, considering both convergence time and accuracy, TW is an optimal solution to the efficient trust assessment problem in large scale VIoT.

## 9. Conclusions

The increasing number of vehicles on the road can be an excellent source for crowd-sourced applications, VIoT, and V2X services. In all of these applications, the authenticity of disseminated information among neighbor vehicles is much needed, as vehicular networks are highly affected by human factors and malicious attacks. Furthermore, previously proposed security solutions are mostly based on public key infrastructure. These traditional solutions much emphasis on security and privacy issues, but ignores the notion of uncertainty and trust management among vehicles in VIoT. Therefore, this paper proposes trust enhances routing TER by taking advantage of the TW algorithm for efficient route discovery. Also, trust assessment is efficiently achieved by the TW algorithm in terms of better execution time in a large vehicular network. A performance analysis is carried out to compare our proposed TER scheme with two other AODV and AASR routing schemes. Experimental results validate the accuracy of our proposed scheme in terms of better throughput, lower packet drop, and the end to end delay. In the future, we will enhance this research by considering more recent attack patterns e.g., Man in the middle attack, Masquerading attack, and so on, and try to establish an algorithm to tackle these attacks in vehicular networks.

## Figures and Tables

**Table 1:** Trust field extension added in proposed TER.

Standard On-Demand Routing	Trust Enhanced Routing
Sending node IP address	Receiving node IP address
Sending node seq number	Receiving node seq number
⋯	⋯
hop count	same
⋯	⋯
Expire time	Expire time
⋯	Positive evidence (trust field)
⋯	Negative evidence (trust field)
⋯	Opinion metric
⋯	Trust update

**Table 2:** Node security level authentication based on trust adjustment rules.

$\Omega_{\mathrm{xz},b}^{k}$	$\Omega_{\mathrm{xz},d}^{k}$	$\Omega_{\mathrm{xz},u}^{k}$	Action
		>0.5	Verify signature.
	>0.5		Distrust a vehicle till expiry time.
>0.5			Trust a vehicle.
≤0.5	≤0.5	≤0.5	Request and verify authentication.

**Table 3:** Simulation parameters in trust enhanced routing.

Examined Protocol	TER
Simulation time	100 (s)
Number of nodes	50
Simulation area	1000 m × 1000 m
Movement model	Random way point
Vehicle speed	0~10 m/s
Transmission range	250 m
Physical link bandwidth	11 Mb
Traffic type	CBR/UDP
Packet size	512 bytes
Connection rate	4 pkt/s
Pause time	5 s
Routing Attacks	Black Hole, On-off attack
Number of malicious nodes	0~25

**Table 4:** Statistics of the Advogato and PGP datasets.

Datasets	Vertices’s	Edges	Ave. Deg	Diameter
Advogato	6542	51,227	19.5	4.83
PGP	10,682	24,315	24	4.52

**Table 5:** Statistics of data-sets into real number values using four trust levels.

Trust Level	Trust Opinion	Trust Value
1	(0.08,0.82,0,0.1)	0.08
2	(0.26,0.64,0,0.1)	0.26
3	(0.63,0.27,0,0.1)	0.63
4	(0.85,0.05,0,0.1)	0.85
